# Total Vaginectomy in a True Hermaphrodite: A Case Report

**DOI:** 10.7759/cureus.40104

**Published:** 2023-06-07

**Authors:** Susmita Das, Uma Rani Swain

**Affiliations:** 1 Obstetrics and Gynaecology, Aster Hospital, Dubai, Dubai, ARE; 2 Obstetrics and Gynaecology, Sri Balaji Action Medical Institute, New Delhi, IND

**Keywords:** ovotestis, gonadal dysgenesis, ambiguous genitalia, vaginectomy, hermaphrodite

## Abstract

A case is reported herein of a true hermaphrodite (TH) with an ovotestis, a uterus, a vagina, and an underdeveloped phallus. The patient was raised by his parents as a male, based on the presence of a phallus with ambiguous genitalia. He started experiencing breast enlargement at the age of 14 and menarche by the age of 17. He was reviewed using ultrasound, magnetic resonance imaging of the abdomen, and karyotyping, and the reports showed evidence of Mullerian structures and 46 XX karyotyping. Based on the preferences of the patient and his parents and their psychological outlook toward the male gender, a total mastectomy, hysterectomy, bilateral gonadectomy, and total vaginectomy were performed. This was followed by reconstruction of the male genitalia and supplemented with male hormone replacement therapy. Accordingly, a TH was assigned a male gender.

## Introduction

A true hermaphrodite (TH) is an individual born with ovaries and testicular tissue. It is a rare variety of intersexual disorders [1] and can present as an ovotestis or an ovary and testes on either side [2]. In the presence of an ovotestis, the testis is always located in the center, and the ovary is located in a lateral location [2,3]. True hermaphrodites have ambiguous genitalia, which can cause significant parental anxiety and create psychological and social problems for patients if the condition is not managed in a timely fashion [4]. In this case, the patient was raised as a male due to a distinguishable phallus. However, when the patient started developing female secondary sexual characteristics, his parents approached the hospital for proper gender assignment.

## Case presentation

A case is reported of a 19-year-old male brought into the hospital with the chief complaint of breast development starting at the age of 14 years, with the onset of menstruation at the age of 17. The patient wanted a definite gender assignment, preferably male.

Clinical history

**Figure 1:**
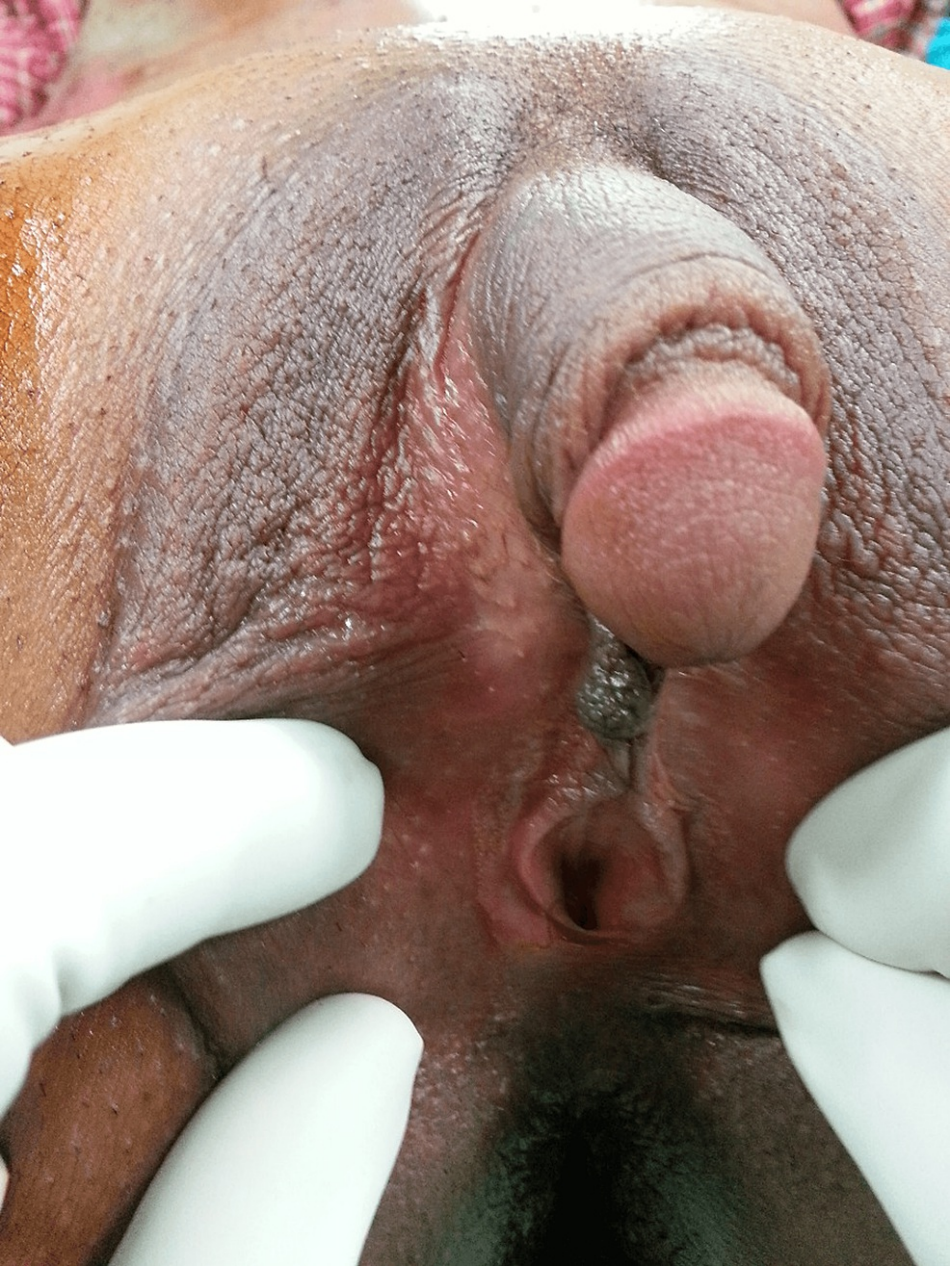
Preoperative picture of the patient with ambiguous genitalia.

The patient was delivered at home without any medical aid. As a child, he had an enlarged phallus without any definite scrotal bulge and was thus raised as a male (Figure 1). At the age of 12, he developed male-pattern hair distribution with further enlargement of the phallus. At the age of 14, breast enlargement was observed, for which his parents consulted a doctor. Based on the patient’s ambiguous genitalia, karyotyping was advised, and he showed 46 XY. However, the patient defaulted on follow-up visits with us for a while.

At the age of 17, the patient began regular menstruation, which led his parents to seek immediate medical attention. Karyotyping was conducted again and showed 46 XX.

**Figure 2:**
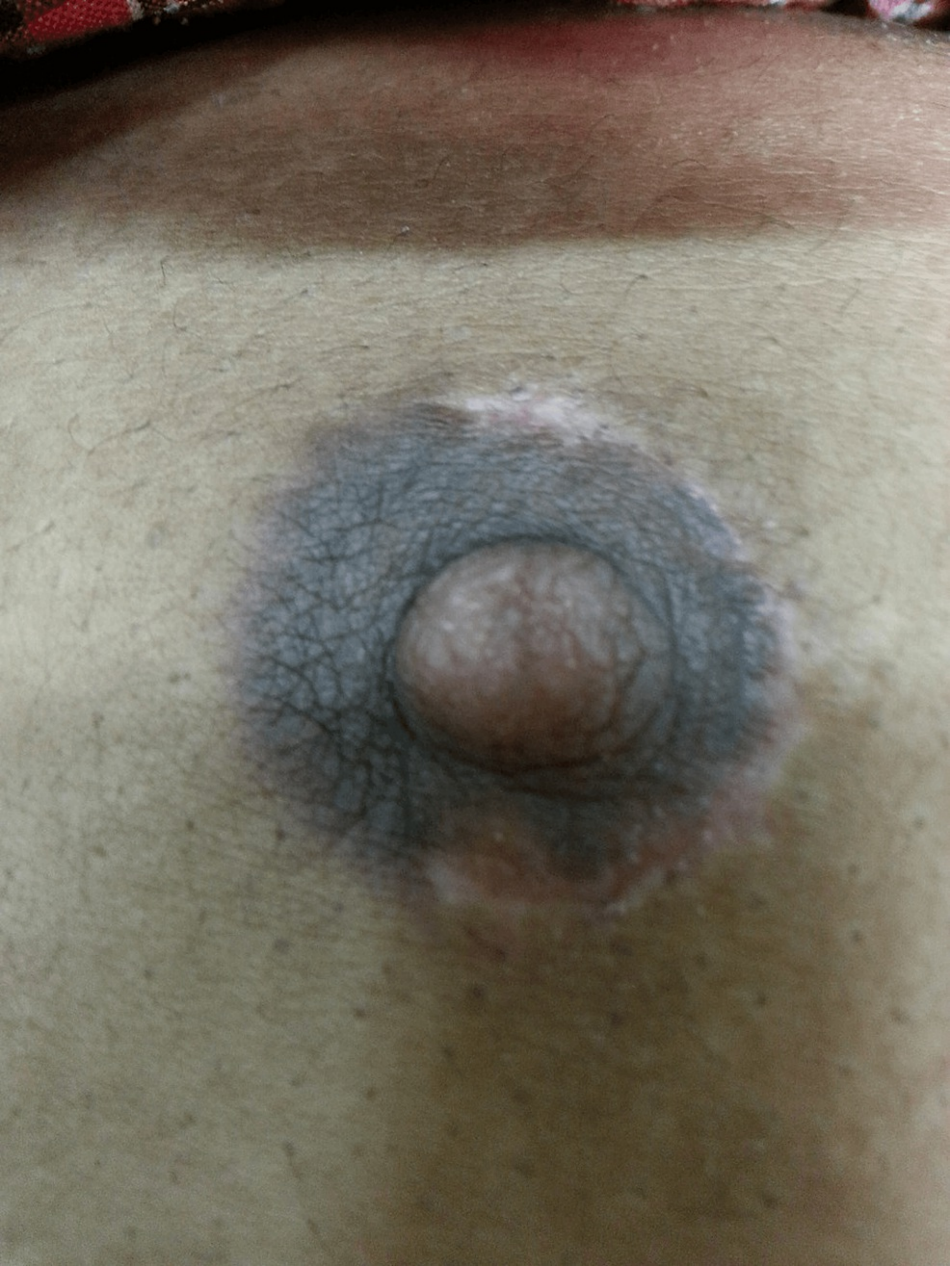
Postoperative image of a subcutaneous mastectomy.

Bringing all the above reports, the patient came to our hospital for a definite gender assignment, preferably male. An ultrasound and magnetic resonance imaging (MRI) were performed for a detailed assessment of the genital organs and to rule out any skeletal or urinary tract anomalies. Both reports confirmed a normal uterus with bilateral normal-sized ovaries. A psychiatric consultation was carried out to declare the patient fit for further gender assignment. The patient was subsequently sent to a plastic surgeon for a bilateral mastectomy (Figure 2) and to endocrinologists for hormone replacement (testosterone) therapy.

Two months after the initial surgery, the patient received a diagnostic laparoscopy, which showed a normal-sized uterus with bilaterally normal-looking fallopian tubes and gonads. The right fallopian tube was not connected to the uterus, but the rest of the abdominal and pelvic tissues were normal.

**Figure 3:**
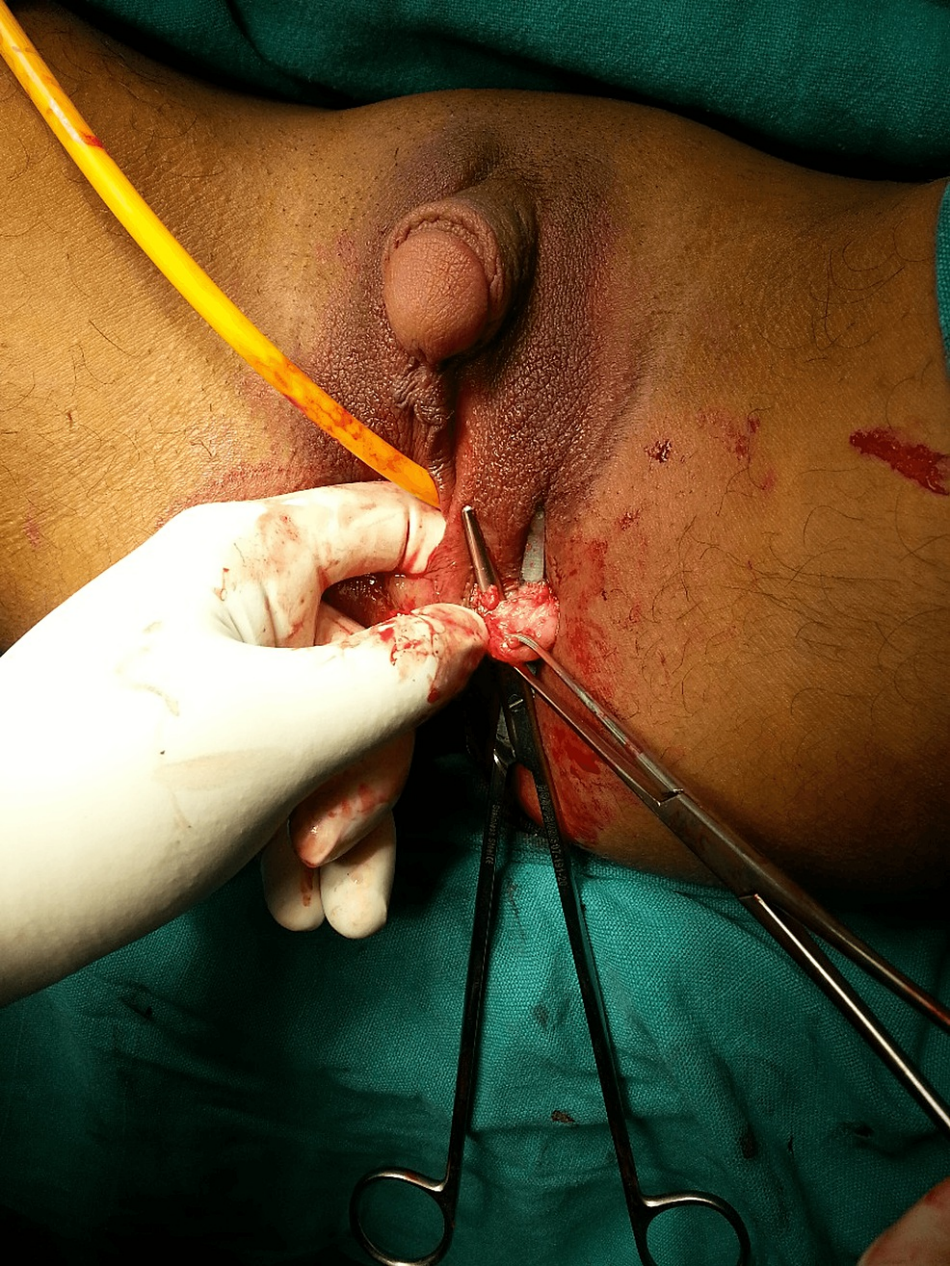
Removal of suspected left testicular tissue.

**Figure 4:**
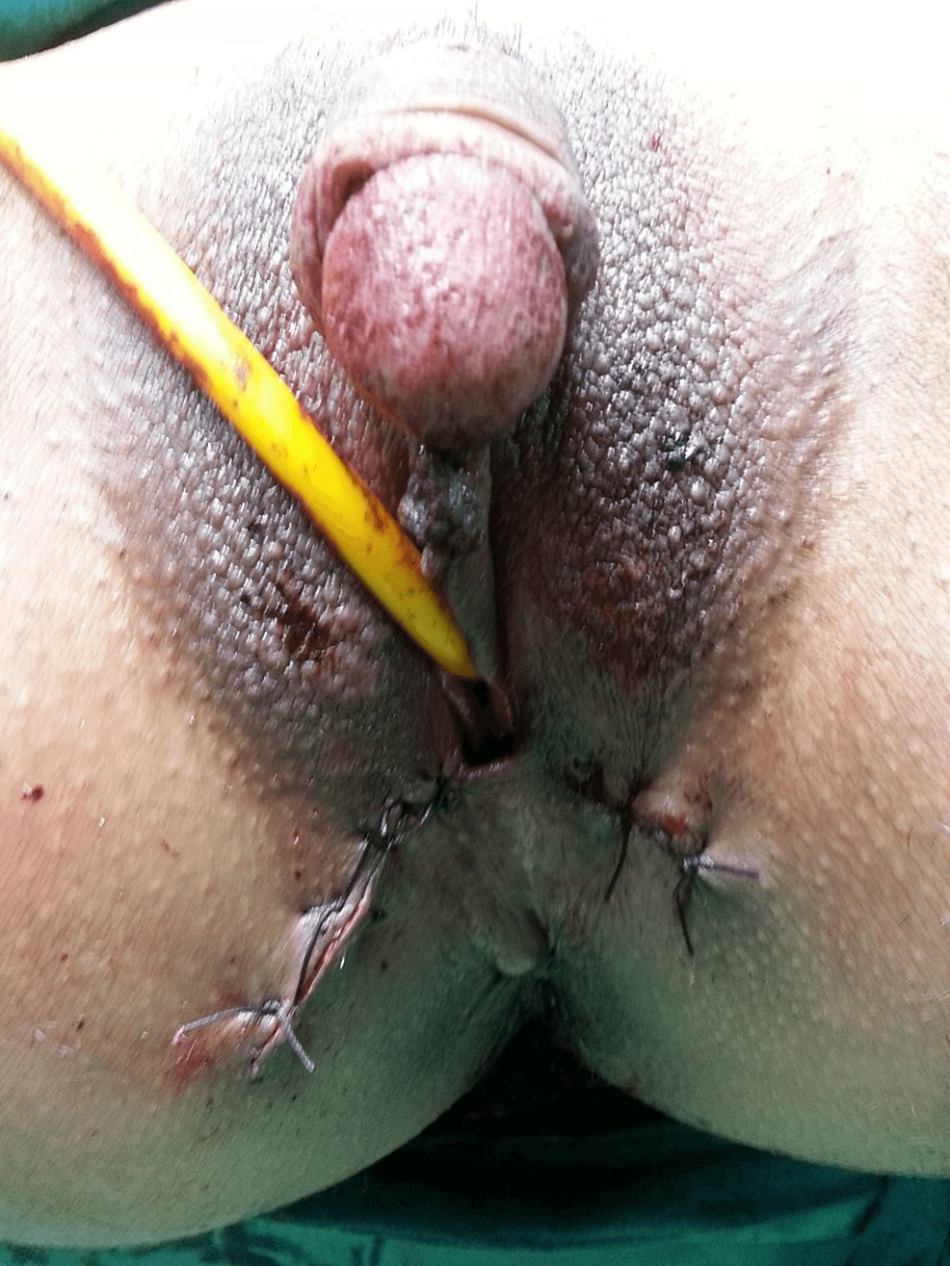
After the laparoscopic-assisted vaginal hysterectomy, a total vaginectomy is performed.

Moreover, a laparoscopic-assisted vaginal hysterectomy with bilateral oophorectomy was performed. Vaginal manipulation was difficult due to the small size of the vagina, and the procedure was performed using a Schuchardt incision on the left side. During this process, small, symmetrical 2 cm × 1.5 cm solid masses were present underneath both sides of the labioscrotal skin (Figure 3). Local excision, assuming these masses to be testis, was done, and all specimens were sent for histopathological examination. This was followed by a total vaginectomy and repair of the perineum (Figure 4).

**Figure 5:**
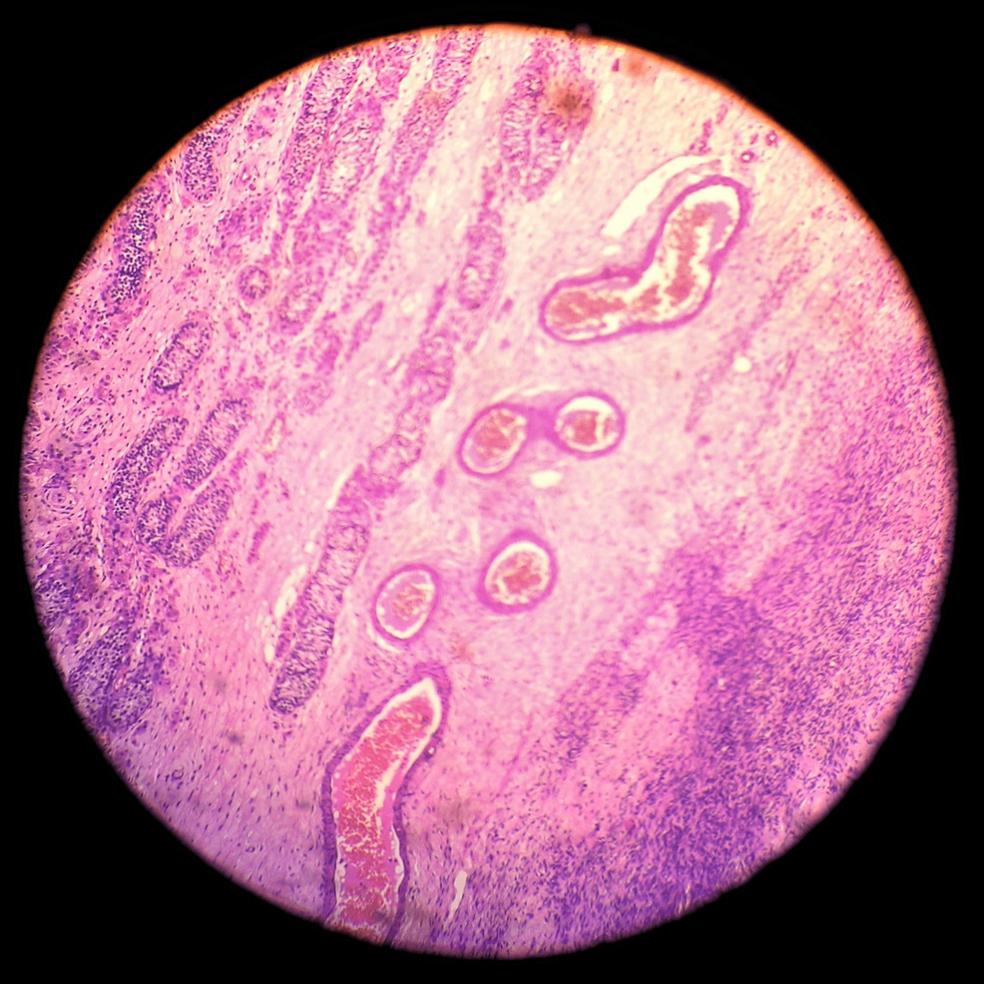
The right ovary includes seminiferous tubules and ovarian stroma, which are separated by fibrocollagenous tissue and blood vessels.

**Figure 6:**
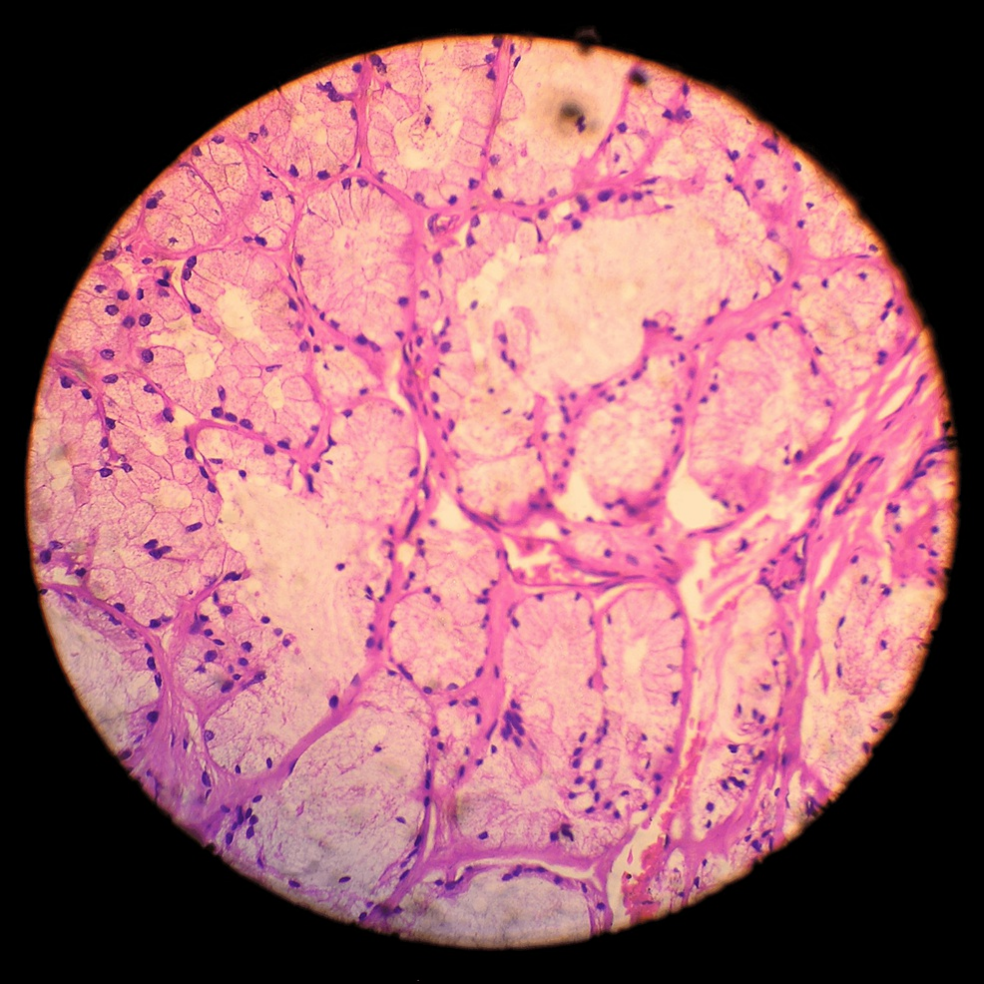
Surgically removed specimen presuming testicular tissue, histologically labeled as Bartholin gland hyperplasia.

The histopathology report showed ovotestes on the right side with well-formed seminiferous tubules, showing germ cells but no sperm (Figure 5). There was also normal ovarian parenchyma with follicles. On the right side, there was a fallopian tube and ductus deferens in close approximation, while on the left side, there were unremarkable tubes and an ovary with a para-tubal cyst. The uterus showed a proliferative-phase endometrium with unremarkable myometrium associated with chronic cervicitis in the cervix. Solid masses removed as bilateral testes were histologically confirmed as Bartholin gland hyperplasia (Figure 6).

The postoperative period was uneventful, and the patient was subsequently referred to a urologist for reconstructive surgery of the scrotum and penis.

## Discussion

Normal fetal sexual development depends on multiple factors, e.g., chromosomal, gonadal, and hormonal. Defects in any of these result in disorders of sexual differentiation (DSDs), an uncommon complex heterogeneous group of conditions. Approximately 5% of DSDs occur in THs [1,2]; however, the exact number of cases is not confirmed, as they are mostly reported from tertiary centers after evaluation.

Various classifications have been used to group DSDs, but the most commonly used classification is that Allen proposed based on histological evaluation [5]. The differentiation between significant groups of ambiguous genitalia-female pseudo-hermaphroditism, male pseudo-hermaphroditism, mixed gonadal dysgenesis, and true hermaphroditism-is based on clinical, biochemical, radiological, and histological evaluation [6].

Most patients are genotypically female (46 XX); other known karyotypes are 47 XXY, 46 XX/46 XY, 46 XX/47 XXY, or mosaicism with Y-bearing cells lying limited to the gonads [7,8].

A TH presents the rarest form of ambiguous genitalia and is characterized by the simultaneous presence of testicular and ovarian tissue types; the condition has an incidence rate of 1/20,000 [9], or 5% of DSDs [1,2].

Typically, THs present as one of three types: unilateral, bilateral, or alternate. The unilateral condition occurs when testicular and ovarian tissue types form testes or ovaries on one side of the body. The case is considered bilateral if testicular and ovarian tissue types are on both sides of the body. Moreover, the case is considered an alternate when a testis forms on one side and an ovary on the other [10].

Approximately 90% of patients have ambiguous genitalia, e.g., a microphallus, hypospadias, urogenital sinus, labioscrotal swelling or fusion, gynecomastia, cryptorchidism, or chordae, and they may present with lower abdominal pain [1]. Most patients typically present with a well-developed vagina as per the age of presentation, and the presence or absence and degree of development of the uterus and fallopian tubes depend on the level of androgens, estrogens, and Mullerian inhibitory factors. The presence of both Mullerian and Wolffian structures in these patients has been explained by the inappropriate timing of adequate Mullerian-inhibiting factors and insufficient synthesis of testosterone by Leydig cells [11]. The patient in the current case, who was initially raised as a male due to having an enlarged phallus, presented with painful breast development, male-type hair growth, and the subsequent initiation of regular menstrual cycles.

In addition to clinical examinations, imaging studies like pelvic ultrasounds, computed tomography scans, MRIs, pelvic studies, and diagnostic laparoscopies can also be used for evaluation. Histologic evaluation of collected tissue samples from patients during surgery can help to determine the sex of the patient, regardless of the karyotype result [12]. Cytomolecular tests are performed to study genetic sex; however, karyotyping is not used to determine the rearing sex of the patient [13,14].

Endocrinological tests for androgen sensitivity assessment and other reproductive hormones should be considered, along with gonadotropin-releasing hormone agonists for females and anti-Mullerian hormone levels for males. These tests will help evaluate the gonadal function by comparing the baselines of aberrant gonadal tissues pre- and post-removal [13,14].

Lai et al. [15] reported a case report of a TH for a 35-year-old male with right cryptorchidism who had had a retractable mass in the left inguinal region for 20 years. A subsequent biopsy suggested true hermaphroditism with seminoma, but the patient was phenotypically male, karyotype (XY), and married with a child.

Samantray et al. [16] reported that a 20-year-old male with left cryptorchidism presented with abdominal pain. A laparoscopy showed Mullerian structures and the left testes near the left inguinal ring. Since the patient was raised as a male, an abdominal hysterectomy and bilateral salpingo-oophorectomy were performed, which revealed a seminoma. Moreover, a biopsy of the right testis was done.

Yahaya et al. [17] reported a five-year-old phenotypically male child clinically presenting with right cryptorchidism. The patient underwent a right orchidectomy and the removal of Mullerian structures.

Along with a diagnosis of TH via confirmed histology, gender assignment is based on predominant phenotypes like fat distribution, the degree of pubic hair distribution, and other correctional surgeries like clitoroplasty and vaginoplasty. It is crucial to discuss with multispecialty health professionals, parents/guardians, psychologists, and patients before the sex of rearing is assigned. The earlier the patient is evaluated, diagnosed, and surgical repair performed (ideally between three and six months), the better it is psychologically for the parents and the child.

In children with cryptorchidism, corrective surgery is recommended within two years of birth; however, if the patient’s age is above the age of two years, undescended testes should be removed and biopsied to rule out malignancy [15].

The patient in the current case study was reared as a male until the age of 14 due to having an enlarged phallus. Once he began experiencing breast development and menstruation three years later, he presented for medical evaluation and gender assignment via correctional surgery.

After understanding the patient's and his parents' mental outlook and preferences, the patient underwent a subcutaneous mastectomy, followed by the removal of ovotestes, the uterus, and fallopian tubes, and finally, a vaginectomy. This was followed by reconstruction of the male genitalia, which was supplemented with male hormone replacement therapy. Thus, a TH was assigned a male gender.

## Conclusions

In the case of sexual differentiation disorders, it is essential to diagnose a patient as being a TH or with mixed gonadal dysgenesis because the appropriate diagnosis is a crucial factor in conserving the gonads. Definite gender assignment is decided based on phenotype, genotype, gonadal histology, rearing gender, and social factors.

In this case, since the patient presented at the late age of 19, importance was given to the gender of rearing and social factors. Such cases should be given the earliest attention to prevent social gender ambiguity.
